# Understanding the genetics of peripartum depression: Research challenges, strategies, and opportunities

**DOI:** 10.3389/fgene.2022.1022188

**Published:** 2022-11-17

**Authors:** Eva E. Lancaster, Dana M. Lapato, Roseann E. Peterson

**Affiliations:** ^1^ Department of Psychiatry, Virginia Institute for Psychiatric and Behavioral Genetics, Virginia Commonwealth University, Richmond, VA, United States; ^2^ Department of Human and Molecular Genetics, Virginia Institute for Psychiatric and Behavioral Genetics, Virginia Commonwealth University, Richmond, VA, United States; ^3^ Department of Psychiatry and Behavioral Health, Institute for Genomics in Health, State University of New York Downstate Health Sciences University, Brooklyn, NY, United States

**Keywords:** peripartum depression, postpartum depression, depression, genetics, women’s mental health, genome-wide association studies, polygenic risk scores, major depressive disorder

## Abstract

Peripartum depression (PD) is a common mood disorder associated with negative outcomes for mother and child. PD is an understudied disorder in psychiatric genetics, and progress characterizing its genetic architecture has been limited by a lack of disorder-specific research, heterogeneous and evolving phenotypic definitions, inadequate representation of global populations, low-powered studies, and insufficient data amenable to large meta-analyses. The increasing availability of large-scale, population-level efforts, like biobanks, have the potential to accelerate scientific discovery and translational research by leveraging clinical, molecular, and self-report data from hundreds of thousands of individuals. Although these efforts will not fully equip researchers to confront every challenge posed by systemic issues in data collection, such as the reliance on minimal phenotyping strategies, the field is in a position to learn from other successful psychiatric genetic investigations. This review summarizes the current state of PD genetics research and highlights research challenges, including the impact of phenotype depth, measurement, and definition on the replicability and interpretability of genomic research. Recommendations for advancing health equity and improving the collection, analysis, discussion, and reporting of measures for PD research are provided.

## 1 Introduction

Pregnancy-associated depression (hereafter referred to as peripartum depression [PD]) is a potentially debilitating mood disorder and one of the most common complications of pregnancy and childbirth. PD partially shares symptoms with the nonpathologic postpartum “baby blues” but is a distinct disorder characterized by greater impairment and longevity. PD episodes impair daily functioning and can present with intense feelings of sadness, guilt, anxiety, anhedonia, disturbed sleep, significant decreases in energy and concentration, and thoughts of death, self-harm, suicide, and infanticide (Wisner et al., 2013). PD affects approximately one in seven women in the United States (US), (Wisner et al., 2013) but global prevalence estimates vary substantially depending on how PD is defined and measured and on population demographics, including socio-economic environment and cultural perceptions of mental health and motherhood (Halbreich and Karkun, 2006). Adverse outcomes associated with PD include increased risks for maternal mortality, poor maternal-offspring bonding, paternal depression, poor maternal self-care (e.g., noncompliance with medical guidance), poor infant feeding outcomes, and increased likelihood of behavioral issues in early childhood (Slomian et al., 2019). Early intervention and treatment can reduce the likelihood of negative outcomes, (Batt et al., 2020) but as many as 50% of PD cases may go unrecognized or undertreated (Payne and Maguire, 2019).

PD genetic studies have the potential to uncover biological mechanisms involved in pathogenesis and advance precision psychiatry efforts by identifying individuals at high risk and those likely to respond to treatment (pharmacogenetics). However, several challenges need to be overcome before robust, generalizable findings can be produced. Researchers are optimistic that the creation and use of large-scale public biobanks and repositories will accelerate scientific discovery and offer solutions to longstanding issues in biomedical research like non-representative cohorts and low statistical power (Guintivano et al., 2019; Kimmel et al., 2020). While the shift to biobanks will alleviate some issues, it is not a panacea. Significant challenges will persist for PD research unless scientists capitalize on opportunities to promote rigorous, inclusive studies through improved study design and community engagement. This manuscript identifies challenges in PD genetics research, discusses how each may impact future meta-analytic efforts, and concludes with recommendations to advance the understanding of PD etiology.

## 2 The genetic etiology of peripartum depression

Twin and family studies have consistently supported the notion that major depression (MD), and by extension, PD, are complex disorders influenced by both genetic and environmental factors (Sullivan et al., 2000). The largest family study of PD to date reported that additive genetic factors account for a higher proportion of variance in PD liability compared to that of non-perinatal depression (54% and 32%, respectively) (Viktorin et al., 2016). In general, sample sizes in PD twin and family studies have been modest, likely because a key requirement for a PD twin study is that both twins in a pair have been pregnant at least once reducing available samples when studying PD compared to MD.

Most of the molecular genetic PD research has focused on candidate gene studies (Batt et al., 2020; Guintivano et al., 2018). Candidate gene studies select and test only a small number of genes or genetic variants (“candidates”) for an association with a phenotype. The candidates are chosen based on assumptions that the products of those genes influence the phenotype through a hypothesized biological mechanism. In PD research, hormone receptors for oxytocin (*OXTR*) and estrogen (*ESR1*) have been popular candidates (Payne and Maguire, 2019). The candidate gene approach has been largely unsuccessful because not enough is known about the human genome to make reasonably good guesses about which variants to test (Border et al., 2019). Given that candidate genes are no more predictive of MD than non-candidate genes, and robust replications have been sparse, it is likely that the majority of reported associations between PD and candidate gene variants represent false positives (Border et al., 2019).

An alternative to testing only a few loci is to test variants across the entire genome *via* genome-wide association studies (GWAS). GWAS offers an agnostic approach for identifying trait-associated variants, and GWAS summary statistics can be used to estimate heritability, quantify shared genetic architecture across traits, and construct aggregated risk scores. In order to robustly detect the expected small effect sizes of common genetic variants, GWAS requires large sample sizes to achieve adequate statistical power (Wray et al., 2012). The largest published PD GWAS to date consisted of 3,804 cases and 6,134 controls, a sample size vastly underpowered to detect genome-wide effects (Kiewa et al., 2022; Wray et al., 2012) and approximately 100 times smaller than the largest published MD GWAS (Levey et al., 2021; Howard et al., 2019).

In order to increase power to detect associations, whole-genome research has primarily relied on aggregate genetic methods to characterize genetic relationships between PD and other traits, often psychiatric disorders. This approach leverages summary statistics from GWAS of related disorders to calculate individual aggregate measures of genetic risk (e.g., summing number of risk variants), known as polygenic risk scores (PRS). The PRS are then applied to predict PD in an independent target sample with the purpose of describing the degree of genetic similarity between PD and the related disorder. While genetic correlation estimates more accurately assess the shared genetic etiology between two disorders, (Bulik-Sullivan et al., 2015) cross-trait PRS analysis does not require that well-powered GWAS are available for both traits of interest. Significant associations between PD and PRS for MD, (Kiewa et al., 2022; Bauer et al., 2019; Pouget et al., 2021; Rantalainen et al., 2020) bipolar disorder, (Byrne et al., 2014; Pouget et al., 2021) and schizophrenia have been reported (Rantalainen et al., 2020). While the estimated SNP-based heritability of PD has remained fairly consistent (h^2^ = 0.22), (Kiewa et al., 2022; Byrne et al., 2014) the magnitude of relationships between PD and PRS for other psychiatric outcomes has varied. For example, Byrne et al. (2014) found that bipolar disorder exhibited the greatest genetic overlap with PD (*R* ^2^ = 1.64%), while other studies have either not replicated this association (*R* ^2^ = 0.01%) (Bauer et al., 2019) or identified stronger relationships between PD and the PRS for MD (PRS-MD *R* ^2^ = 7.6%) (Kiewa et al., 2022). These inconsistencies are likely influenced by cross-study differences in study design choices, including phenotyping, sample collection, and statistical power (Bauer et al., 2019).

## 3 Defining and measuring peripartum depression

### 3.1 Defining PD

**FIGURE 1:**
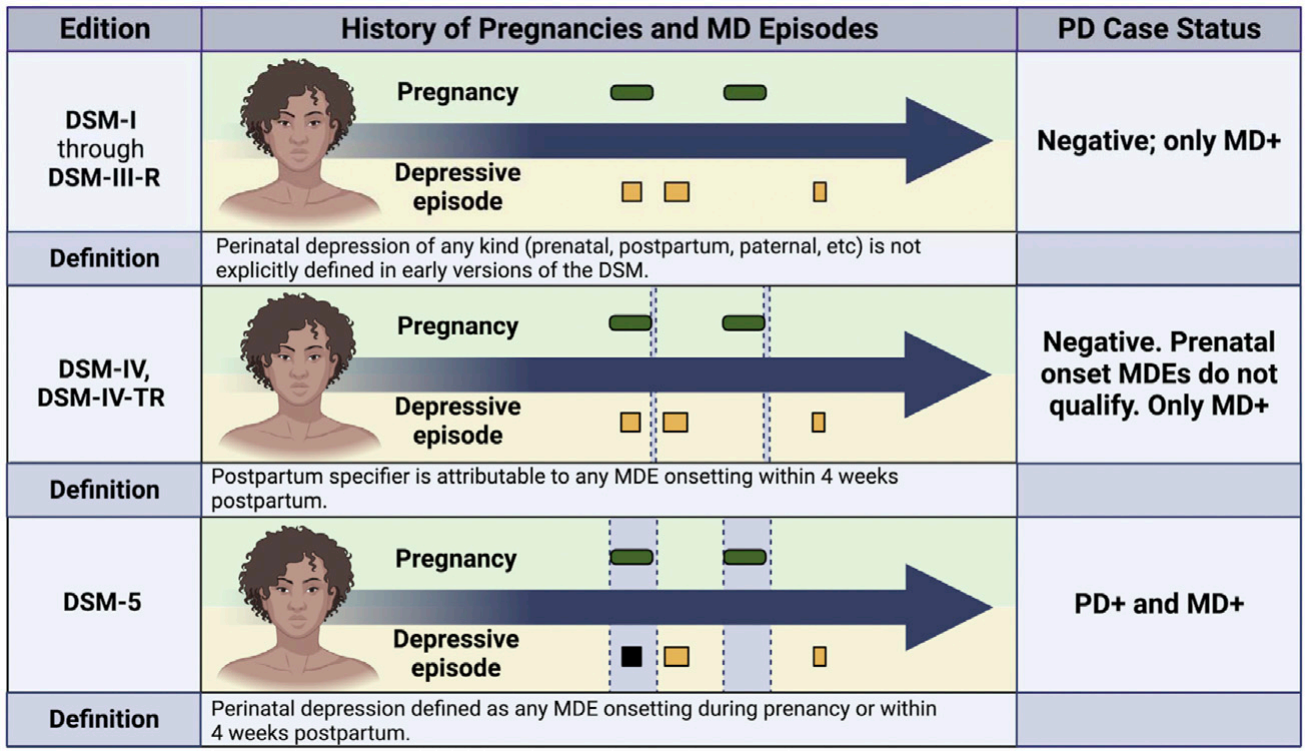
**Impact of Phenotypic Definition on Peripartum Depression Case Status.** This figure illustrates how the same medical history would be classified in a case-control study of peripartum depression (PD) based on the evolving criteria for diagnosis that have appeared in the Diagnostic and Statistical Manual (DSM). For each subpanel, green ovals indicate pregnancies, and squares indicate depressive episodes. Black squares indicate depressive episodes that meet diagnostic criteria for PD, and yellow squares indicate depressive episodes that do not meet PD diagnostic criteria. The diagnostic window for each definition is denoted by vertical grey boxes in the center section. For DSM-IV, the narrow vertical grey box represents the 4 week window following pregnancy when the postpartum specifier would be applied to MD episodes onsetting during that time period. The diagnostic window is much larger for DSM-5 and includes all of pregnancy. Note that before 1994, researchers would have had to apply their own definition because no specifier or diagnosis was included in the DSM before DSM-IV. MD, major depression; MDE, major depressive episode; PD, peripartum depression; DSM, Diagnostic and Statistical Manual.

Arguably one of the most challenging aspects of studying PD is the evolving nature of its clinical definition (Figure 1) which has complicated epidemiological efforts to estimate prevalence and study trends over time. Pregnancy-related depression was not distinguished by specifiers or a unique diagnosis in the Diagnostic and Statistical Manual (DSM) until 1994 when it appeared as a postpartum specifier to MD in the DSM-IV (American Psychiatric Association, 1994). The most recent edition (DSM-5) expands the definition of PD to include any major depressive episode that onsets either during pregnancy or within the first 4 weeks postpartum (American Psychiatric Association, 1994). The International Classification of Diseases code (ICD) recently underwent a similar expansion from ICD-10 and ICD-11, to include the prenatal period and up to 6 weeks postpartum (World Health Organization (WHO) 1993; World Health Organization (WHO) 2019). While this shift in clinical definition reflects improvements in prenatal care, the need to harmonize data across multiple diagnostic definitions complicates meta-analyses and increases difficulties in drawing cross-study comparisons (Rantalainen et al., 2020; Bauer et al., 2019).

### 3.2 Measuring PD

A challenge related to but distinct from defining PD is measuring it. A variety of measurement strategies exist, and choice of measurement tool(s) directly impacts the cost of research and the interpretability of the results. Broadly speaking, psychiatric disorders can be assessed by diagnostic thresholds or by symptom-based measures. Symptom-based measures typically correlate with clinical diagnosis status and may index disorder severity, but they are not tantamount to clinical diagnoses, which typically yield greater heritability estimates and smaller prevalence estimates (Kendler et al., 2019; Foley et al., 2001). The most commonly used measurement tool in PD research is the Edinburgh Postnatal Depression Scale (EPDS), a validated self-report questionnaire measuring PD symptoms (Cox et al., 1987). The EPDS was designed to prioritize mood symptoms over somatic symptoms (e.g., feelings of sadness versus changes in weight) to prevent physiologically normal changes in weight, appetite, and energy that are typical during the peripartum from inflating the depressive symptom count. The questionnaire is short, simple to administer, and sufficiently accurate as a clinical screening tool (sensitivity and specificity estimated as 85% and 84%, respectively, using a cut-off of 10) (Levis et al., 2020). The EPDS is validated, but not in every language or culture. As psychometric studies suggest that the accuracy of EPDS scores varies across countries, languages, and cultures, it may not be an ideal instrument for research outside of majority English-speaking countries (Halbreich and Karkun, 2006).

Minimal phenotyping (sometimes called broad or shallow phenotyping) refers to using short, often non-validated questionnaires to collect self-reported details about demographics, medical history, disorder status, and more. An example of minimal PD phenotyping would be the use of a single question asking, “Have you ever been diagnosed with perinatal depression? (Yes/No)” to determine PD case status. This phenotyping strategy would not collect details about the episode (e.g., timing of onset, duration, and severity) or a comprehensive account of the participant’s history of MD and PD. In contrast, deep phenotyping includes assessment tools like structured clinical interviews, which are the most accurate psychometric instruments for evaluating psychiatric disorders. Clinical interviews generate rich, reliable data but are time-intensive and costly to perform. Thus, they may be prohibitively expensive or infeasible to collect for large-scale studies. In general, the more measures collected, the greater the financial cost and accuracy of the data. A compromise between minimal and deep phenotyping is to use one or more validated self-report questionnaires and/or medical record abstraction.

### 3.3 Impacts of minimal phenotyping

Recent research exploring the impact of minimal phenotyping on efforts to characterize the genetic architecture of MD has found that using broadly-defined health outcomes instead of rigorously-defined phenotypes can produce misleading and non-specific results (Cai et al., 2020). Broad MD phenotypes derived from minimal phenotyping have significantly different genetic signatures from clinically-defined MD to the extent that some minimal MD phenotypes have greater genetic correlations with other psychiatric disorders than clinically-defined MD does (Cai et al., 2020). In other words, minimal MD phenotypes appear to index general psychopathology rather than MD-specific psychopathology (Kendler et al., 2019; Cai et al., 2020). These consequences primarily stem from the fact that the minimally phenotyped samples do not actually represent clinical MD (Kendler et al., 2019; Cai et al., 2020). Minimally defined MD cases (including PD) represent an aggregation of several separate phenotypes, including patients meeting clinical criteria for MD, participants with subclinical levels of depression, misdiagnosed participants who have related but distinct condition(s) (e.g., bipolar disorder), individuals scoring high for neuroticism, and patients prescribed antidepressants to treat off-label conditions (Kendler et al., 2019). This phenotypic heterogeneity obscures association signals and is a plausible explanation for why MD defined by shallow phenotyping is less heritable (SNP-based heritability of 11%–13% compared to 26%) and more genetically related to other psychiatric disorders than stricter clinical definitions of MD (Cai et al., 2020). In addition to contributing to challenges in identifying trait-specific molecular pathways, low resolution data also decreases the odds that responses and medical records will be comparable since the ancillary details (e.g., timing of episode onset in the case of PD) are not available to facilitate harmonizing data from multiple sources.

An analogous depth of phenotyping study has not been performed for PD because sufficient data does not exist. PD is underrepresented even in large, EHR-based population cohorts like All of Us (All of Us Research Program Investigators et al., 2019). As of 6 June 2022, the All of Us study included 372,380 participants. Of the 227,740 participants with EHR data, 1.4% had a diagnosis of schizophrenia (population prevalence of 1%) compared to 0.2% with postpartum depression (prenatal and peripartum depression are not listed). This underrepresentation echoes and is partially driven by PD going unrecognized in clinical settings. Even without the data necessary to perform a replication study, it is reasonable to assume that the findings from the MD depth of phenotyping study apply to PD because the phenomenon of genetic associations changing with respect to disorder definition has been observed for other disorders, particularly those at risk for frequent misdiagnosis. A 2022 review on the progress of Alzheimer’s GWAS research noted that heritability estimates had fallen from 17% to 3% when the cohorts transitioned from using clinically assessed Alzheimer’s disease cases to “proxy dementia cases” despite an increase in sample size (Escott-Price and Hardy, 2022). The high degree of clinical, and likely etiological, heterogeneity in complex disorders like MD and Alzheimer’s disease, may further exacerbate the consequences of minimal phenotyping in genetic research.

## 4 Recommendations for future research

Although genetic research into PD is behind that of other psychiatric phenotypes, (Guintivano et al., 2018) the field is in a position to learn from genetic investigations into related outcomes. While the characteristics unique to PD must be considered, progress in the characterization of the genetic architecture of MD could be used to inform the best approach for identifying biologically meaningful associations in PD research. Careful consideration must be paid to study design and data collection to avoid amplifying non-specific signals, increase the opportunity for data harmonization, provide evidence for pertinent questions about the distinctiveness of PD compared to MD, and to ensure that results are broadly applicable across diverse cultures and ancestries (Table 1). Transparent reporting and adherence to open science practices will improve chances that studies can be meta-analyzed appropriately and aid in interpretation, particularly when comparing results across studies.

**TABLE 1:** Recommendations for the advancement of PD genetics research.

Reporting		
Action	Impact	Recommendation
Equating clinical diagnoses and symptom-based measures	Conflates phenotypes with potentially distinct genetic architectures	Specify what is being measured
		Consistently refer to phenotype as symptoms or diagnosis
		When referencing published studies, specify the phenotype used by the cited studies and acknowledge if the finding/relationship applies to both symptom-based and diagnosis-based phenotypes
**Data Collection**		
**Action**	**Impact**	**Recommendation**
Recording only PD case status	Limits data reuse	Record details about timing of episode onset and symptoms to maximize data reuse
Recording MD case status without specifying if pregnancy-associated MDE	Limits data reuse and ability to separate MDEs by clinical context and reduce heterogeneity	Record details about timing of onset of all MDEs, pregnancies, and births
**Health Equity**		
**Action**	**Impact**	**Recommendation**
Data collection without stakeholder engagement	Miss important perspectives, community input, and engagement	Meaningful engagement of stakeholders from diverse patient groups and communities
		Invest in community-based participatory research
		Commitment from funding agencies to invest in community and stakeholder partnerships
Excluding non-European ancestry data from genetic analyses	Reduces generalizability and limits precision psychiatry	Apply ancestry inclusive methods (e.g., trans-ancestry GWAS mixed models)
Ignoring important environmental and social determinants of health	Supports incomplete etiological models and exacerbates health disparities	Collect important environmental measures (e.g., socioeconomic status, adverse childhood experiences)

Abbreviations: GWAS, Genome-wide association study; MD, major depression; MDE, major depression episode; PD, perinatal depression.

### 4.1 Reporting

Comprehensive reporting, particularly on the timing of episodes and types of measures, can improve data harmonization, both by creating more opportunities for meta-analysis and assisting in interpreting results across studies. It remains essential to use accurate language to describe the periods surrounding pregnancy and childbirth (e.g., peripartum, prenatal, postpartum), so that depressive episodes occurring during pregnancy are clearly distinguished from depression outside of the peripartum period. Clarifying pregnancy-associated terms is especially pertinent as PD definitions have shifted over time to include episode onset during pregnancy (i.e., from DSM-IV to DSM-5 and ICD-10 to ICD-11; Figure 1) (World Health Organization, 1993; American Psychiatric Association, 1994; World Health Organization, 2019). Effort should also be made to avoid conflating PD symptoms with diagnoses. The majority of whole-genome studies of PD rely on symptom-based screening instruments, such as the EPDS, to measure PD (Byrne et al., 2014; Rantalainen et al., 2020; Pouget et al., 2021; Kiewa et al., 2022). These tools are used either as a means to determine case status by setting a symptom threshold or to test relationships between genetic factors and symptom load as a continuous measure. Given that distinct genetic architectures have been identified for clinical diagnoses of MD and depressive symptom measures, (Kendler et al., 2019) equating a clinical diagnosis of PD with symptom-based measures may introduce additional noise and reduce power to detect PD-specific genetic effects. Researchers should clearly and consistently distinguish between symptom-based and diagnosis-based phenotypes, and may consider evaluating results from studies examining PD symptoms separately from those using clinical diagnoses. In some cases, studies may collect more details than they share (e.g., collecting symptom-level data but sharing only case-control status), resulting in low resolution data and decreasing the odds that the data will be comparable. Openly sharing detailed accounts of all available measures with associated summary statistics can help address these challenges.

### 4.2 Data collection

Considering that a well-powered GWAS of PD has yet to be published, increasing sample collection is a primary concern. However, larger sample sizes at the cost of phenotypic precision may not be the only or best strategy to detect PD-specific genetic effects (Escott-Price and Hardy, 2022). The China, Oxford and VCU Experimental Research on Genetic Epidemiology (CONVERGE) consortium identified the first robust genome-wide associations with MD despite having fewer samples than other large-scale efforts by focusing on deeper phenotyping and reducing the clinical heterogeneity of the sample (Major Depressive Disorder Working Group of the Psychiatric GWAS Consortium et al., 2013; CONVERGE Consortium, 2015; Peterson et al., 2018). Similarly, PRS generated using strictly-defined MD had the same predictive ability as broadly-defined MD but in smaller sample sizes (Cai et al., 2020).

Several attributes of PD require the need for accurate and comprehensive data collection. As PD is a subtype of MD, more detailed data collection may be necessary to understand the potentially subtle differences in variation between PD and MD. Collected cases need to be clearly distinct from both MD occurring outside of the peripartum and from women experiencing the “baby blues” to minimize the possibility of misclassification. Considering that the PD diagnostic criteria has changed over time (Figure 1), detailed longitudinal collection will allow for continued data harmonization and improve data reuse even if definitions continue to evolve. Details beyond PD case status, such as the timing of any MD episodes, pregnancies, and births, will need to be recorded to verify whether individuals continue to meet criteria. If following current diagnostic codes, the window of time postpartum to meet criteria for PD is relatively small (Figure 1) and may also benefit from prospective data collection. Finally, given the interest in identifying PD-specific processes and understanding how they may differ from MD etiological mechanisms, (Kiewa et al., 2022; Bauer et al., 2019; Byrne et al., 2014; Pouget et al., 2021) comprehensive information about participants’ history of depression and other comorbid psychiatric disorders should be collected.

### 4.3 Health equity

A disproportionate majority (>78%) of large-scale GWAS of major psychiatric disorders are conducted in European ancestry cohorts (Peterson et al., 2019). Indeed, the largest GWAS of PD only included European ancestry individuals (Kiewa et al., 2022). The underrepresentation of diverse ancestral populations in research seriously limits the understanding of disease etiology. Historically, traditional GWAS approaches have required ancestral homogeneity among study populations, which has excluded ancestrally admixed individuals and underrepresented ancestral groups in order to reduce spurious findings from effects of population stratification. However, emerging trans-ancestry methods are increasingly available and should be applied to be as inclusive as possible (see Table 1) (Peterson et al., 2019). Given that large-scale PD data collection efforts are required, the field and funding agencies have an opportunity to make deliberate data collection choices. Inclusive recruitment strategies will require significant time devoted to trust building and community engagement such as in community-based participatory research (Cargo and Mercer, 2008). In addition to community engagement, research teams should include diverse perspectives and lived experiences including expertise in cross-cultural psychiatry as well as members from historically underrepresented groups in research (Hofstra et al., 2020).

Large-scale psychiatric genetic studies have largely ignored important environmental risk factors in their models despite known effects. This practice limits the understanding of disease and leads to incomplete etiological models. Including environmental risk factors can aid in detecting genetic associations by accounting for heterogeneity in risk. For example, in MD, additional genetic risk variants were uncovered by stratifying participants in the GWAS by severe adversity exposure (Peterson et al., 2018). Similarly, data collection efforts should include metrics of social determinants of health (e.g., access to prenatal care, social support) in order to identify interactions with genetic risk and points for intervention for improved health outcomes. Race and ethnicity are socially-derived categorical constructs that can provide information about social factors that affect risk for disease, including having a lived experience of social injustice. However, they are often inappropriately used as a proxy for genetic ancestry which is a biologically-derived continuous construct. Researchers should clearly distinguish race from ancestry in order to tease apart specific biological, psychological, and social determinants of health. Broadening ancestral diversity of studied populations coupled with investigations of social determinants of health will improve the effectiveness of genomic medicine by expanding the scope of known genomic and environmental risk and resilience factors towards advancing the understanding of PD disease etiology.
